# Manganese exposure assessment in formula-fed infants in Israel

**DOI:** 10.1186/s13584-025-00688-2

**Published:** 2025-04-15

**Authors:** Jonatan Darr, Ziva Hamama

**Affiliations:** Food Risk Management Department, The National Food Service, Ministry of Health, Jaffa St. 236, Jerusalem, Israel

**Keywords:** Infants, Infant-Formulas, Manganese, Exposure-assessment, Israel, Nutrition

## Abstract

**Background:**

Proper nutrition is fundamental to the regular mental and physical development of infants, toddlers, and children. Overexposure to manganese (Mn) in infants has been correlated to various behavioral and neurological symptoms such as lower IQ, attention deficit hyperactivity disorder, and impairment in fine motor skills. The following study aims to evaluate exposure to Mn in formula-fed infants in Israel from birth to nine months of age.

**Methods:**

Over 200 infant formulas of multiple brands were sampled by the Israeli National Food Service, as part of a routine monitoring of levels of various nutritional components, including Mn. Data on levels of Mn in water was drawn from routine monitoring programs carried out by the Ministry of Health (MOH). Total energy requirements were calculated based on current infant weight and growth data collected over the past decade in MOH-operated family care centers. Dietary exposure was assessed for infants from birth to six months as the sum of Mn intake from infant formula and potable water. For infants aged seven-nine months, Mn intake from complementary feeding was assessed based on national surveys of feeding behavior in infants aged nine-twelve months.

**Results:**

Milk-based infant formula brands consistently demonstrated lower levels of Mn compared to other formulations. Almost half of the sampled formula brands exceeded regulatory tolerance to deviation from labelling of nutritional components. Though some variation in Mn concentrations is evident in water sources across Israel, the overall contribution of water to Mn intake is negligible given the high levels of desalination in Israel. Excessive Mn intake in formula-fed infants is evident across multiple formula brands.

**Conclusions:**

When breastfeeding is not optional, milk-based formulas are the most suitable in terms of their relative contribution to Mn intake. Equating maximal levels of Mn in potable waters to levels set in EU and USA regulations is advisable. A greater regulatory tolerance for deviation from labelling of mineral content is advisable so as not to hinder importation of infant formulas.

**Supplementary Information:**

The online version contains supplementary material available at 10.1186/s13584-025-00688-2.

## Background

Proper nutrition is critical in maintaining the health and wellbeing of the general population [1–3]. It is particularly fundamental in infants, toddlers, and children, where it promotes normal mental and physical development, and reduces the life-long risk of developing chronic diseases [2, 4]. Developmental, physiological, and genetic factors affect the nutritional requirements of an individual [5–7], and these requirements change with age, habits, and health [8–10]. The nutritional needs of infants and toddlers are distinct to those of adults, which is reflected, among others, in the recommendations of both the World Health Organization (WHO) and the Israeli Ministry of Health (MOH) to exclusively breastfeed infants during the first six months of life [11].

Despite these recommendations, upwards of 17% of Israeli infants exclusively consume infant formula [12], and from the age of two months this figure increases to 27%. At four months, only 33.7% of infants are exclusively breastfed. These numbers are similar to those published by the WHO, which reports that worldwide less than half of the infants younger than six months old are exclusively breastfed [13]. The relative high prevalence of infant formula consumption demands attention to the nutritional intake of formula-fed infants.

From around the age of six months, infants should be introduced to food other than breast milk or infant formula, and their nutritional uptake gradually shifts to mimic the nutritional habits of their caretakers [14]. This shift includes gradual habituation to solid foods and drinking water that are consumed in growing quantities as the child grows. Yet, the nutritional demands of infants and toddlers remain different to those of their parents and older siblings. Several physiological differences contribute to these unique nutritional demands, including, for instance, the ongoing development of the central nervous system [15, 16].

Manganese (Mn) is a trace element naturally found in food and drinking water and obtained predominantly from these sources. Mn serves as a coenzyme in multiple biological pathways, including metabolism of carbohydrates and amino acids and scavenging of reactive oxygen species in the mitochondria [17]. Though symptoms associated with Mn deficiency are rarely described, Mn toxicity is known to occur, and affects mostly the nervous system [18, 19].

Occupational overexposure to Mn dust, for example through welding and mining activities, has been linked to various health effects involving the central nervous system [20, 21]. Toxic exposure leads to physiological and psychiatric symptoms known as manganism. These symptoms include muscle spasms, mania, depression, and delusion. With chronic overexposure, symptoms can progress to neuromotor impairments similar to those associated with Parkinson’s disease, including changes in gait and balance, tremor, and rigidity.

In addition to the symptoms associated with toxic exposure to Mn, multiple studies found that chronic overexposure in children is significantly associated with adverse neurodevelopmental outcomes [22–26]. Chronic overexposure to Mn in children, through drinking water and air-born pollution, was correlated with lower scores on intelligence tests and various behavioral problems including Attention Deficit Hyperactivity Disorder (ADHD), when compared to children with lower levels of Mn exposure.

Mn absorption following dietary ingestion may be higher in infants and children compared to adults who absorb only about 1–5% of dietary Mn [27, 28]. This possibility, along with the reported long-terms effects of chronic overexposure in children, necessitates an evaluation of the dietary intake of Mn from birth to adulthood. The following study aims to evaluate the dietary exposure to Mn relative to available Dietary Reference Values (DRVs) in formula-fed infants from birth to the age of nine months, and the compliance of water and infant formulas to the regulatory requirements in Israel.

We demonstrate that Mn intake in formula-fed infants is higher than current Adequate Intake (AI) recommendations, and that there is a high probability of exceedance of the WHO recommended Tolerable Daily Intake (TDI) of 25 µg/Kg_BW_. The formula brand and base formulation are key factors in Mn intake, whereas the contribution of potable water to the total intake is negligible, given the measured Mn levels.

## Methods

### Infant formula and mineral water sampling

Between Jul. 2020 to Sep. 2023, 205 imported infant formula samples were collected at port during routine risk-based monitoring by inspectors of the National Food Service (NFS), and analyzed by the Haifa Public Health Laboratory for mineral content. Compositional data on 127 additional samples from different batches, representing 12 local brands, were transmitted to the NFS following a request addressed to local infant formula producers. These were internal compliance tests conducted by the companies to verify that batches meet required specifications and have not been validated by external labs. Finally, 28 samples of bottled mineral water were collected from the local market during routine monitoring programs by NFS inspectors and analyzed for Mn concentrations.

### Manganese measurements

Mn concentrations in infant formula and mineral waters were measured using inductively coupled plasma-mass-spectrometry (ICPMS) based on the EPA 200.8 method for determination of trace elements in water and wastes. Internal standards were used to compensate for analytical errors.

### Manganese concentration in tap water

To assess the relative contribution of Mn in potable tap water to the total dietary exposure, data from routine monitoring surveys carried out between 2018 and 2023 by the MOH was collected and analyzed for Mn. This dataset included 1068 samples from 708 different water sources scattered across the country, representing raw waters drawn from aquifers, surface water, or water pumped into desalination plants.

Desalination effectively removes any Mn from the raw waters pumped into the facilities. An average of 70% of the tap water in Israel undergoes desalination, with a maximum of 80% of the supply in some regions originating in desalination [29]. As such, exposure to Mn from potable tap water results from the remaining 30% of water. Weighted average of Mn concentrations in raw waters not pumped into desalination plants was calculated to estimate the contribution of water that has not undergone desalination.

### Estimations of infant growth rate in Israel

In order to evaluate infant growth in Israel, a total of 9,026,360 weight records, taken between 2013 and 2024, from 1,417,372 male and female individuals aged zero-nine months, were extracted from data collected by MOH-operated family care centers. From these, a total of 6,956,084 records, representing single monthly measurements were used to estimate average daily caloric intake. In addition to weights, data regarding bottle-feeding habits was drawn from previously published surveys carried out in these centers [12].

### Daily kCal and water intake assessment

To estimate the consumption of infant formulas, we referred to the 2001 Food and Agricultural Organization (FAO) expert panel assessment report regarding human energy requirements [30]. Total energy requirements were calculated using the data drawn from MOH-run family care centers on weights of male and female infants aged zero-nine months. Estimations were calculated for the 3rd, 50th, and 97th percentiles. Mean values across males and females were used. In addition, it was considered that newborns normally lose no more than 10% of their body weight up to a week after birth, with full recovery within approximately two weeks after birth [31]. To estimate the mean water intake, the range of labelled kCalories (kCal) per 100 ml was taken from the sampled brands.

### Manganese exposure from complementary feeding

To assess exposure to Mn from complementary feeding, we used data from the Mabat infant national health and nutrition survey of 2019–2020, conducted to estimate the nutritional behavior of infants aged nine-twelve months [32]. Mean Mn concentrations of the relevant food items included in the survey were extracted from the United States Department of Agriculture (USDA) nutrition database [33]. To estimate the Mn intake from complementary feeding in six-nine month-old infants, the kCaloric intake from complementary feeding in infants nine-twelve months old, was scaled down to 50% of the kCaloric intake of infants aged six-nine months old. This, since by the age of nine months an average of 50% of the nutritional needs of a growing infant should be supplied from complementary foods [34].

### Statistical analysis

Statistical analysis was performed with SPSS version 25 statistic software package. Comparisons between groups were performed of non-parametric tests. A value of *P* < 0.05 was considered as statistically significant unless otherwise indicated.

## Results

### Manganese levels in infant formulas

**Fig. 1:**
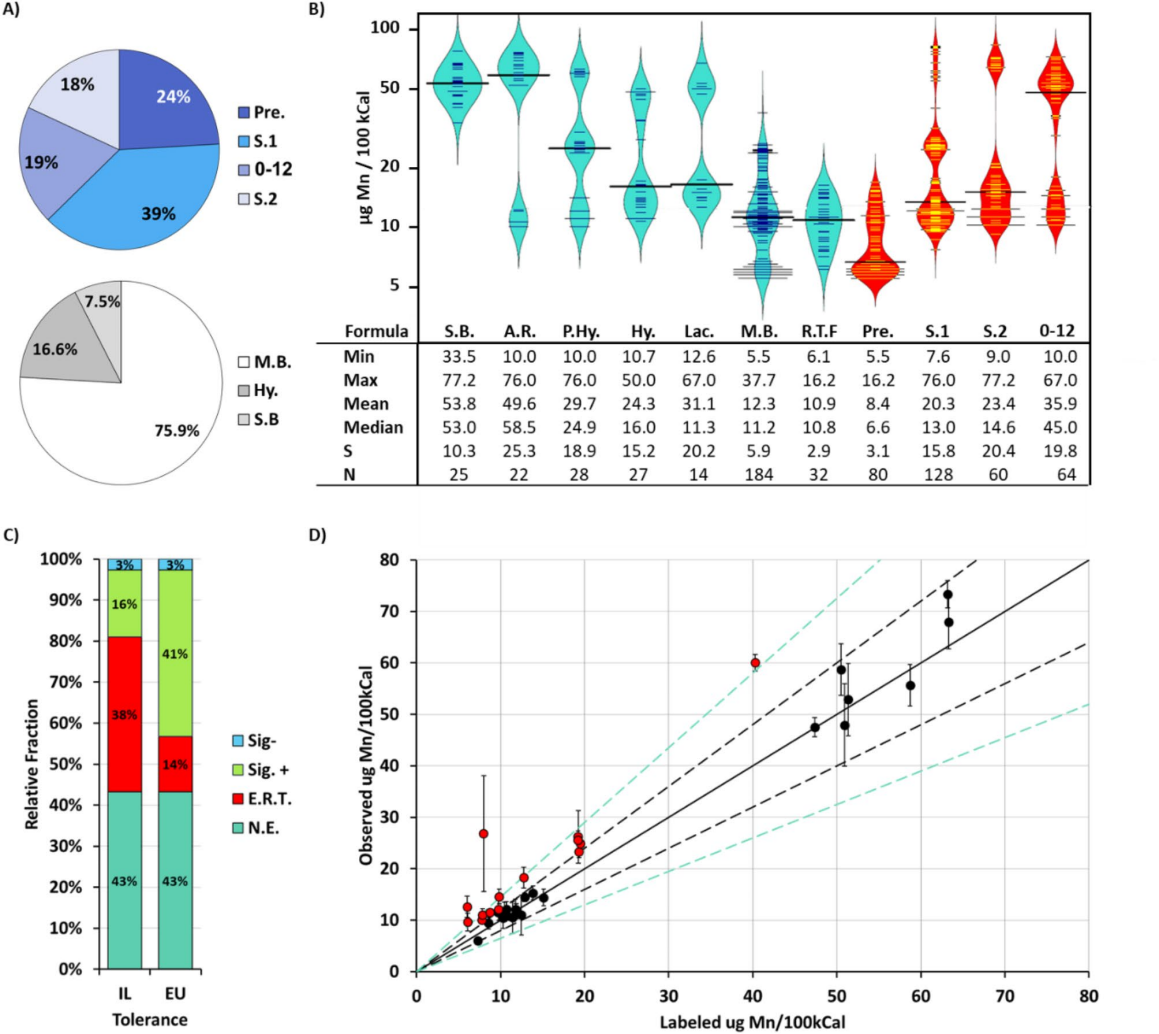
Mn concentration and compliance with label. **(A)** Relative percent of each category of sampled formula. S.B. = Soy-Based; A.R. = Anti-Reflux; P. Hy. = Partially Hydrolyzed; Hy. = Hydrolyzed; Lac. = Lactose Reduced / Free; M.B. = Milk-Based; R.T.F. = Ready to Feed; Pre. = Pre-term; S.1 = Stage 1; S.2 = Stage 2 **(B)** Bean plot distribution and summary statistics of Mn levels in sampled formulas per 100 kCal. An independent Kruskal-Wallis test demonstrated statistically significant differences in means and in distribution of Mn level across formulations with a *P* < 0.001. **(C)** Relative fraction of compliant and non-compliant formula brands. E.R.T = Exceeding Regulatory Tolerance; Sig.+: Statistically higher compared to label; Sig.-: Statistically lower compared to label. N.E. = No Exceedance; Two-sided T-test, *P* < 0.05. **(D)** Labelled vs. observed Mn concentrations. Significant exceedance of regulatory tolerance is evident in the red labelled data points, Two-sided T-test, *P* < 0.05. Dashed line = Israeli regulatory tolerance. Dashed green line = EU regulatory tolerance

Of the 332 infant formulas included in this study, 76% were milk-based, 16.6% Hydrolyzed / Partially hydrolyzed, and the rest were soy-based (Fig. 1A). In addition, 39% of sampled formulas were stage one (for infants from birth to six months), 24% were for premature infants, and 18% were stage two (for infants six-twelve months old) (Fig. 1A). Additional details are available in Supplementary Table 1.

Mn levels in all sampled formulas were above the required minimal levels of 1 µg/100kCal and below the guidance upper levels of 100 µg/100kCal (Fig. 1B), as set by the Directive on Composition Requirements and Nutritional Requirements of Formulas Intended for Infants and Toddlers [35] and the relevant Codex Alimentarius standard [36].

Mean and median Mn concentrations varied between different types of formulas based on the formulation type and / or the brand (Fig. 1B). The lowest mean and median Mn concentrations were measured in ready-to-feed formulas labelled for preterm infants at 8.4/6.6 µg/100kCal. The highest mean Mn concentration was measured in soy-based formulas at 53.78 µg/100kCal, whilst the highest median concentration was measured in anti-reflux formulas at 58.42 µg/100kCal.

The degree of variability was markedly higher in anti-reflux, lactose reduced/lactose free, and hydrolyzed formulations compared to milk-based, soy-based, and ready-to-feed formulations, in which the degree of variability was lower (Fig. 1B).

Consumer loyalty to brands, and especially to infant formula brands, is high both globally and in Israel [37, 38]. As such, consumers are not expected to change formula type frequently, if at all. In order to estimate the dietary intake of Mn from formulas, brands were grouped based on the type of formula (milk-based, plant-based, etc.) and stage (premature, stage one, stage two etc.), and the range of Mn concentration was calculated for each category based on the mean measured Mn concentration per brand. The range is provided in supplementary Table 2.

### Compliance with labelling requirements

Out of the 37 brands sampled, in 56.7% (21 brands) the mean Mn levels were observed to be significantly different from the labeled concentration (Fig. 1C), this without taking into account the measurement uncertainty. However, the Public Health Protection Regulations (Food– Nutritional Labelling) 5778 − 2017 allows for a tolerable deviation of ± 20% from nutritional labelling given natural variations in the composition of raw materials. With this in mind, only 38% (14 brands) of formulas significantly deviated from the labelled concentration (Fig. 1C-D). In a few brands, where the average Mn concentration was found to be within the tolerable range, isolated batches were evident in which the measured Mn level exceeded the regulatory tolerance range. Tolerable deviation in Israel is lower compared to other jurisdictions. In the European Union (EU) the tolerable deviation for minerals from their labelled concentrations is + 45%, -35%. Under these standards only 14% (5 brands) were found to deviate (Fig. 1C-D).

### Mn levels in drinking water

Powdered infant formulas must be rehydrated with potable water to prepare the ready-to-feed formula. Mn may be present in potable water in various concentrations and contribute to the overall exposure to Mn. To estimate the level of Mn in potable tap water, we drew data from routine water surveys conducted by the MOH between 2018 and 2023.

**Fig. 2:**
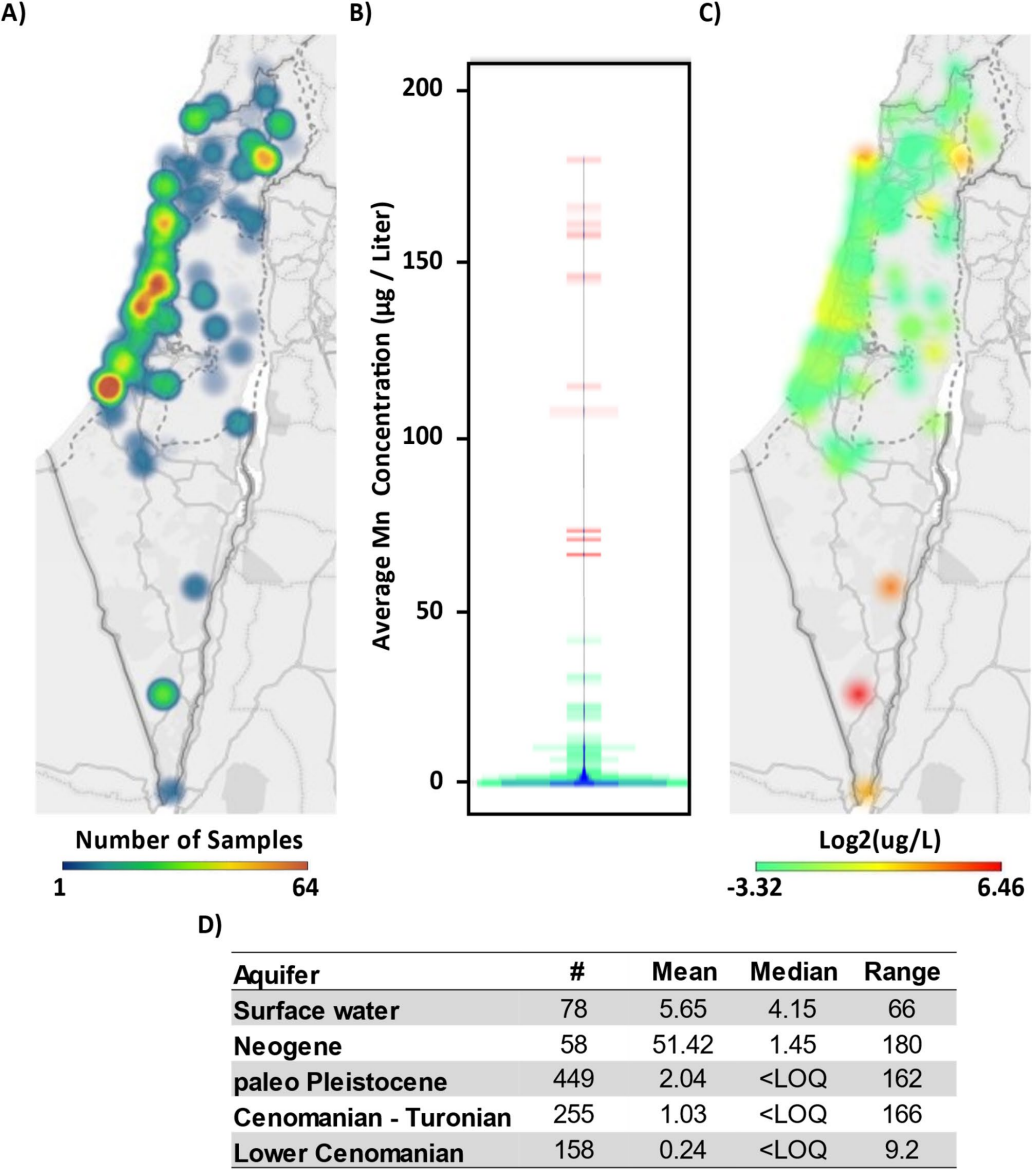
Mn concentrations in raw waters. **(A)** Geographical distribution of raw water sampling frequency between 2018–2023. **(B)** Bean plot of average Mn Concentration of raw water sources that are not desalinated in µg/l. Green < 50 µg/L; Red > 50 µg/L. **(C)** Log2 transformation of Mn concentrations in raw waters. **(D)** Mean, median and range concentrations of Mn based on the source of water. Values are given in µg/l

The majority of sampled raw water were from drills along the Mediterranean coast, pumping stations in the Galilee Sea, and pumping stations in desalination plants (Fig. 2A). During this period only two samples out of 1068 (0.187%) exceeded the maximal level of 200 µg/liter set under the Public Health Regulations (Sanitary Quality of Drinking Water and Drinking Water Facilities) 5773 − 2013. Additionally, only 1.8% (13 of 708) of raw water source not intended for desalination plants were above the 50 µg Mn/liter limits for potable water set in the EU and the USA (Fig. 2B).

Mn concentrations demonstrated a large spatial variability, related to the source of the waters (Fig. 2C-D). The highest concentrations of Mn were measured in raw water from drills to Neogene aquifers in the south of Israel that provide brackish waters for desalination plants. A few additional isolated wells across the country demonstrated incidental cases of high Mn levels. The highest median levels, of 4.15 µg/L, were measured in surface waters pumped from the Galilee Sea. Based on these Mn surveys, and given that roughly 70% of the tap water in Israel originate in desalination plants [39], the average concentration of Mn in tap waters is estimated to be 0.25 µg/L.

As the Israeli MOH recommends using boiled tap water or boiled bottled mineral waters for the preparation of infant formulas, mineral waters from local and imported brands were also tested for Mn concentration. Mn levels were below the Limit Of Detection ( LOD, 0.05 ug/L) in all local mineral waters sampled.

### Manganese intake in formula-fed infants from birth to six months

Mn intake can be evaluated in exclusively formula-fed infants using the measured Mn concentrations in infant formulas and potable waters (described above) and the estimated caloric intake and estimated water intake (as presented in supplementary Fig. 1A-B).

**Fig. 3:**
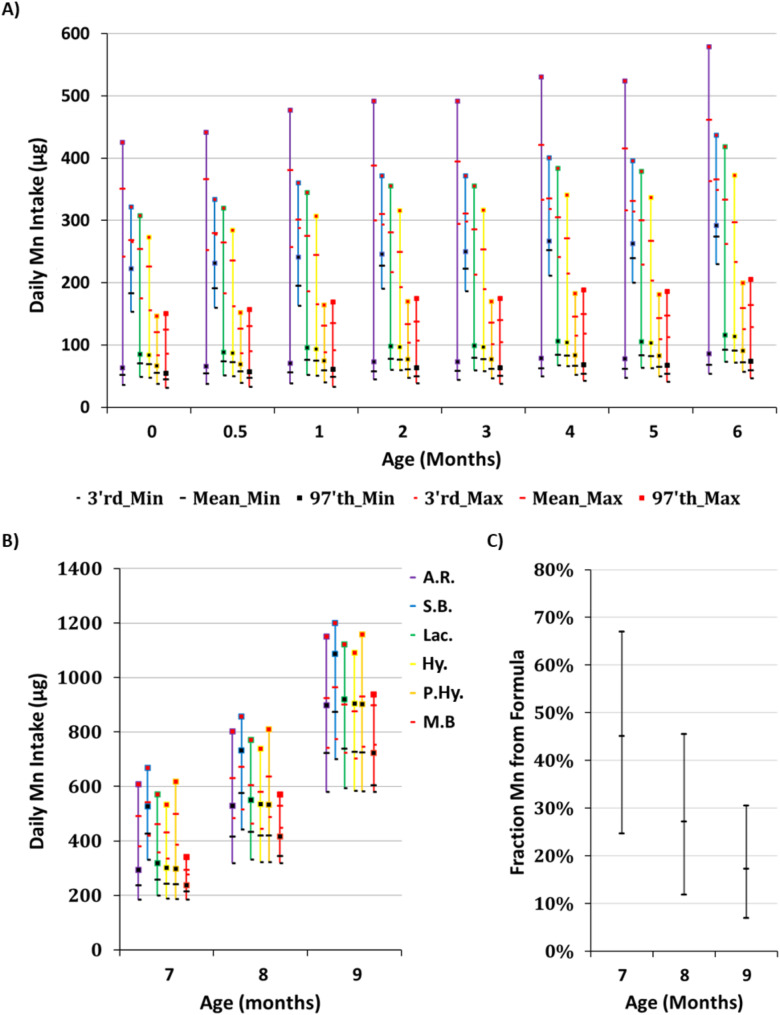
Estimated range of Mn daily intake in infants aged 0–9 months old. **(A)** Estimated daily Mn intake from rehydrated ready to feed infant formula, in infants aged 0–6 months old. **(B)** Estimated daily Mn intake from infant formula, complementary feeding, and water, in infants aged 7–9 months old. **(C)** Estimated relative fraction of Mn intake from infant formula alone in infants aged 7–9 months old. A.R. = Anti-Reflux; S.B. = Soy-Based; Lac. = Lactose Reduced / Free; Hy. = Hydrolyzed; P. Hy. = Partially Hydrolyzed; M.B. = Milk-Based

Total Mn intake in formula-fed infants is estimated to be between 35 µg/day to upwards of 0.5 mg/day for infants aged zero-six months (Fig. 3A). given the low estimated concentration of Mn in potable water in Israel, the total contribution of water to Mn intake is negligible, and the intake is rather highly dependent on the formula brand.

### Manganese intake in infants aged six-nine months old

The MOH recommends gradual habituation to solid foods and drinking water from the age of six months, as does the WHO. Therefore, to estimate Mn intake in children between six-nine months of age, complementary feeding must be taken into account. To this end, we utilized published data from the Mabat Infant National Health and Nutrition Survey [32]. Based on the results of this survey, total caloric intake is estimated to be roughly 930kCal/day in infants aged nine-eleven months, with 70% derived from complementary foods. The Mn intake from complementary foods alone in this age group, is estimated at 1.77 mg/day on average (Supplementary Table 3).

Scaling the intake from complementary foods to comprise no more than 50% of the caloric needs of infants aged six-nine months, total Mn intake from ready to feed formulas and complementary foods can be estimated to range in-between 0.2 mg/day to upwards of 1.2 mg/day (Fig. 3B). The relative fraction of exposure to Mn from infant formula at these ages gradually decreases as weaning progress with the majority of Mn exposure resulting from the complementary food (Fig. 3C). Exposure from potable water is again negligible, given the low concentration of Mn in potable water in Israel.

### Estimated intake relative to dietary reference values

The current recommendations for Mn consumption in Israel can be found in the National Dietary Reference Intakes published by the division of nutrition in the MOH. These are based on the recommendation of the Food and Nutrition Board of the Institute of Medicine (IOM) [40]. For infants up to six months old the adequate intake (AI) of Mn is 3 µg per day, which is based on historic measurements of Mn concentration in breast milk [41–43]. Based on the presented estimations, Mn intake is well above the AI in all infants exclusively fed with formula in Israel, irrespective of the formula brand. In infants fed anti-reflux formula intake can reach as high as 200 times the AI.

For Infants aged seven-twelve months the IOM set an AI of 0.6 mg Mn per day. In contrast, the European Food Safety Agency (EFSA), derived AI levels that range between 0.02 and 0.5 mg Mn per day for infants aged seven-eleven months [44]. Based on our estimations, infants in Israel aged seven-nine months consume between 0.2 and 1.2 mg Mn per day, suggesting sufficient intake that is similar to the estimations reported by EFSA.

**Fig. 4:**
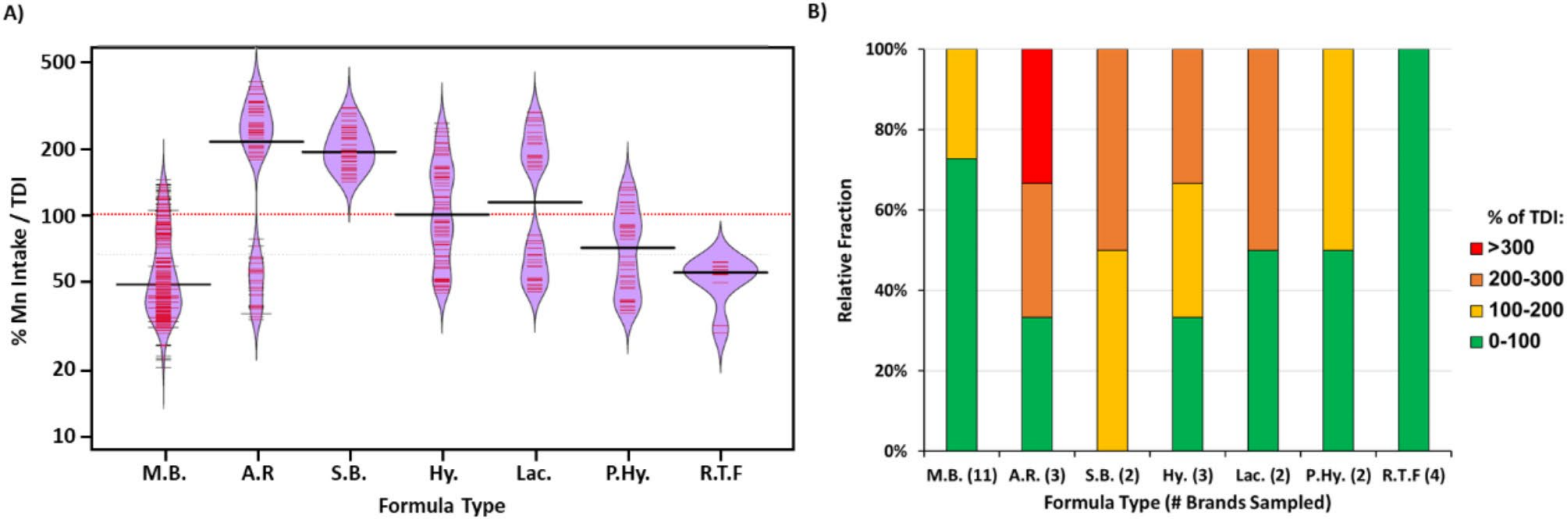
Estimated Mn intake relative to WHO TDI in multiple exposure scenarios. **(A)** Bean plot of estimated Mn intake relative to Tolerable Daily Intake (TDI) for 3rd, 50th, and 97th percentiles of infants aged 0–6 months old, per formula type. **(B)** Relative fraction of formula brands whose consumption by infants up to 6 months old would result in exceedance of WHO TDI of 25 µg/Kg_BW_ per day. Shown by formula type. M.B. = Milk-Based; A.R. = Anti-Reflux; S.B. = Soy-Based; Lac. = Lactose Reduced / Free; Hy. = Hydrolyzed; P. Hy. = Partially Hydrolyzed; R.T.F = Ready-To-Feed

In contrast to AI levels, for the purpose of setting guidelines for drinking water quality the WHO derived a Tolerable Daily Intake (TDI) level for Mn of 25 µg/Kg_Bw_ per day, based on a conservative estimation from animal-derived Lowest Observed Adverse Effect Level (LOAEL) [27, 45]. Relative to this TDI, higher intake is evident in multiple scenarios and multiple ages (Fig. 4A). Indeed, excessive intake can reach as high as 4 times the TDI in new-born infants fed on anti-reflux formulas. The only instance where excessive intake is not evident is with new-born infants fed on ready to feed formulas intended for premature infants.

Evaluation of all the surveyed infant formula brands intended for infants younger than six months, suggested that Mn intake would not exceed TDI in roughly half of the brands (12/23, 52%) (Fig. 4B). Whilst surveyed milk-based brands offer the largest variety of brands in which excessive intake is not anticipated, excessive intake is evident in all surveyed soy-based brands.

## Discussion

Overexposure to Mn in infants has been correlated to various behavioral and neurological symptoms including lower IQ, ADHD, and more [22–26]. As such, evaluation of dietary Mn intake in infants is critical for the protection of public health. To the best of our knowledge, this is the first study that aims to evaluate dietary intake levels of Mn in formula-fed infants in Israel, and compared to previous studies of the topic in other countries, sampling is much more extensive [46, 47].

The Israeli directive on Composition Requirements and Nutritional Requirements of Formulas Intended for Babies and Toddlers [35] sets a minimal level of 1 µg/100 kCal Mn, and a guidance upper level of 100 µg/100 kCal, following the recommendations of Codex Alimentarius standard for infant formula and formulas for special medical purposes intended for infants [36]. These recommendations are also adhered to in the EU delegated regulation 2016/127 [48, 49]. In contrast, in the USA, only a minimal level of 5 µg/100 kCal is set [50].

Israel is heavily reliant on import of infant formulas, and a variety of brands and formulations are routinely sampled by the NFS for various micronutrients, including Mn, following a routine risk-based monitoring of infant formulas at port upon importation. The results of this routine sampling are presented herein (Fig. 1A). Additional release test data was transmitted from local manufacturers following a request by the NFS. All formulas meet regulatory guidelines of minimal limit and guidance upper limit for Mn in infant formulas. Mean, median, and variance levels are highly variable between brands, with milk-based formulas generally demonstrating lower concentrations and lower variability compared to soy-based formulas (Fig. 1B), in line with findings reported in previous studies.

38% of infant brands, and more specifically imported brands, exceed the Israeli regulatory tolerance of ± 20% for the deviation from labelling of nutritional content (Fig. 1C-D) without taking into consideration the measurement uncertainty. Compared to the EU, the allowed regulatory tolerance in Israel is narrow. Moreover, unlike EU policy, the allowed deviation in Israel addresses all nutritional components, be they macro or micronutrients, and regardless of the actual analytical measurement uncertainty of the component. In addition, shelf-life nutrient stabilities vary, but their concentration should remain within tolerable deviation by the end of the shelf life. This introduces an additional consideration in setting an allowed deviation that impacts shelf life and may lead to excessive food loss with significant economic consequences.

When applying the EU tolerance of + 45%, -35% from labelling of mineral content, the infringement rate drops to 14%. Given that the measurement uncertainty is different between various nutritional components, that the stability of nutritional components throughout the shelf life of the product is varied, and considering the limited availability of local production of infant formulas that leads to a high dependence on import of infant formulas, and specifically ones for special needs, we recommend equating tolerance to regulatory tolerance in major trade partners such as the EU.

The MOH should recommend the Israeli parliament to amend the regulations for the protection of public health (food) (nutritional labeling), 2017–5778, so as to allow for a greater regulatory tolerance in Israel that takes into account the analytical measurement uncertainty and stability of different nutritional components. This amendment can promote free trade of infant formulas whose local manufacturing is limited in scope.

Though some geographical variability is evident, Mn concentrations are well below the limit of detection in the majority of raw water samples (Fig. 2). Mn levels were below detection level also in local bottled mineral waters. As such, the contribution of water to Mn intake is negligible. However, local variation is evident and may greatly increase total intake. Maximal levels of Mn in drinking water in Israel should therefore be lowered and equated to 50 µg/L as set in the EU and USA.

Limits for Mn in potable drinking waters in Israel [51] are higher compared to the EU and the USA, where upper concentration for Mn were set to 50 µg/L [52, 53]. As only 2% of sampled raw waters from sources that do not undergo desalination exceeded 50 µg/L (Fig. 2), lowering the upper limit will not hinder the exploitation of existing water sources but may aid in protection of infants in isolated cases where tap water is not of mixed sources and prevent the potential for high intake of Mn in the future.

Total Mn Intake is estimated to be between 0.035 mg/day to upwards of 0.5 mg/day for formula-fed infants aged zero-six months, and between 0.2 mg/day to upwards of 1.2 mg/day for infants aged six-nine months, for whom intake is higher due to the introduction of solid foods (Fig. 3). These intake estimations are well above published adequate intake levels and exceed the WHO tolerable daily intake value of 25 µg/Kg_bw_ per day (Fig. 4). This estimated exceedance is dependent upon the consumed brand and is particularly evident in babies fed with soy-based and anti-reflux formulas.

In contrast to the WHO, the IOM did not establish an upper limit for Mn intake in infants due to lack of sufficient data on the adverse effects in this age group and despite the concerns regarding the ability of infants to handle excessive amounts of Mn. Similarly, the EFSA noted that available data is insufficient to establish an upper limit for infants, despite human and animal studies that support neurotoxicity as a critical factor in Mn overexposure [54].

The excessive Mn intake described herein for formula-fed infants in Israel is exclusively driven by the composition of the formula, with levels exceeding adequate intake and tolerable daily intake levels. Importantly, ingestion is not the only route of Mn exposure. Inhalation and absorption of Mn can contribute to the total exposure of an individual to Mn in addition to ingestion. Our study does not attempt to quantify the total cumulative exposure to Mn that, from all possible routes, may be much higher than our findings reported here.

Mn concentrations in breast milk have been characterized in multiple studies [55] and are considerably lower than the levels observed in infant formulas. However, the bioavailability of Mn, and possibly the bioavailability of additional trace elements, may be considerably higher in breast milk compared to infant formula [56, 57], possibly due to binding of Mn to various proteins found naturally in breast milk [58].

Given that the excessive intake levels of Mn in formula-fed infants are exclusively driven from Mn concentrations in infant formulas, it would be beneficial to set upper limits that better protect infants from the potential neurological effects described in the literature. In Israel, upper Mn levels in infant formulas are aligned to Codex standards. In order to avoid placing an import barrier and a resulting shortage of infant formulas on the Israeli market, the MOH should advise with relevant Codex Committee on Nutrition and Foods for Special Dietary Uses on the results of this study, so as to reconsider international standards for infant formula composition.

Upper levels should be reconsidered based on the most recent scientific data on the bio-availability of Mn in infant formulas and aim towards international harmonization. Data gaps on the bioavailability of minerals in breast milk compared to infant formulas should be addressed by the scientific community to allow regulators to set more appropriate upper limits. In addition, a survey of minerals in breast milk in Israel should be considered, so as to explore the possibility of any local variations and differences compared to published data.

Additional minerals that are normally present in potable water are expected to be depleted in desalinated water that are a major source of potable tap water in Israel. Indeed, magnesium deficiency was observed in the Israeli population following desalination [39]. Additional studies need to evaluate the intake of minerals in formula fed infants in Israel in light of the high desalination rates, so as to assure adequate intake for proper growth, development, and prolonged health benefits.

### Recommended policy actions

The MOH should continue to encourage breastfeeding when and if possible, during the first six months of life.

The MOH should share the results of this study with the Codex Committee on Nutrition and Foods for Special Dietary Uses and actively participate in the setting of standards in the field within the committee discussions.

The MOH should recommend the parliament to amend labelling requirement to allow for a greater deviation from labelling of micronutrients, in line with EU practices.

The MOH’s Committee on the quality of drinking water should recommend the parliament to lower upper Mn levels in drinking water to 50 µg/liter to better protect formula-fed infants.

The MOH should promote a survey of micronutrients in breast milk, possibly in collaboration with the Israeli breast milk bank.

## Conclusions

Breast milk is generally considered as the most balanced diet for infants. As such, breastfeeding is highly recommended during the first six months of life. However, as breastfeeding alone is not always optional, the MOH recommendations state that milk-based formulas are the preferable choice. The results of this study support these recommendations in terms of Mn intake. To better protect formula-fed infants from Mn overexposure, scientific data gaps on the bioavailability of Mn from infant formulas need to be addressed, and an international collaboration is required to re-examine the global standards set for infant formulas based on the most current scientific evidence.

## Electronic supplementary material

Below is the link to the electronic supplementary material.

Supplementary Material 1

Supplementary Material 2

## Data Availability

The datasets used and/or analyzed during the current study are available from the corresponding author on reasonable request.

## Abbreviations

ADHD: Attention Deficit Hyperactivity Disorder
AI: Adequate Intake
EFSA: European Food Safety Agency
EU: European Union
IOM: Institute of Medicine
Mn: Manganese
MOH: Ministry Of Health
NFS: National Food Service
TDI: Tolerable Daily Intake
USA: United States of America
WHO: World Health Organization
