# Supplementary material for: Manganese exposure assessment in formula-fed infants in Israel
Source: Isr J Health Policy Res. 2025 Apr 15;14:24. doi: 10.1186/s13584-025-00688-2 (PMC12001741; doi:10.1186/s13584-025-00688-2)
Supplement: Supplementary file 2 — Supplementary Material 2 [file 13584_2025_688_MOESM2_ESM.docx]

**Supplementary Data:**

**Supplementary Fig. 1: Daily intake in infants from birth to 9 months.**

**
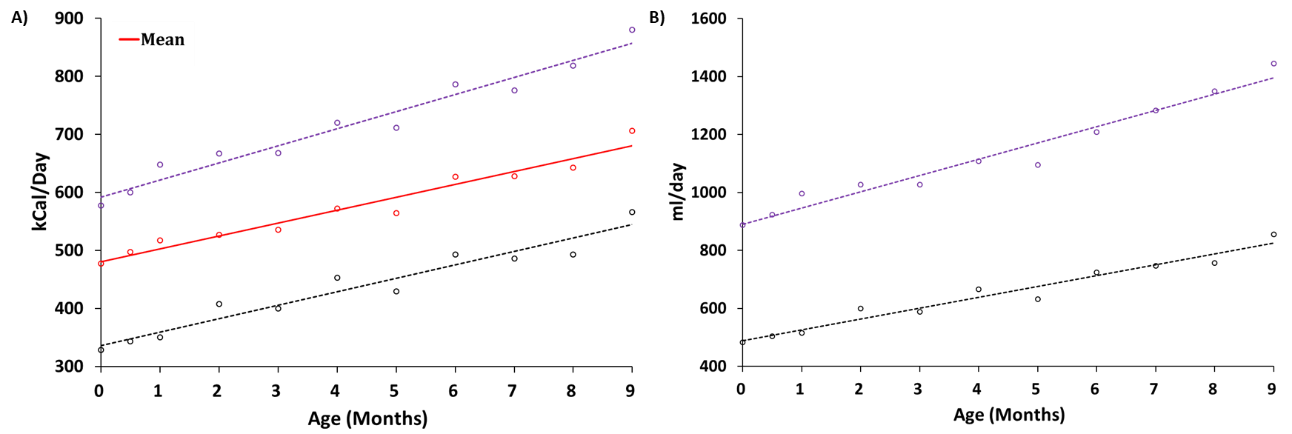
**

**Supplementary Fig. 1: A)** Estimated total kCAl needs per day. Purple = 97'th percentile: y = 29.481x + 591.92, R² = 0.9582; Red = Mean: y = 22.219x + 480.4, R² = 0.9544; Black = 3'rd percentile; y = 23.174x + 335.81, R² = 0.9345. **B)** Total water intake per day in milliliters. Purple = 97'th percentile: y = 56.104x + 890.73, R² = 0.9598; Black = 3'rd percentile; y = 37.397x + 487.92, R² = 0.9553. Intake from birth to six months is from the infant formula in the ready to eat form, following the reconstitution of the powder in portable water. From six months onwards, infants begin supplementing their diet with limited amounts of water or additional beverages.

**Supplementary Table 1:** **Characteristics of sampled formulas**

| **Formula** | **Age** | **# Brands** | **# Samples** | **Companies** |
| --- | --- | --- | --- | --- |
| R.T.F. | Preemie | 4 | 32 | 2 |
| M.B. | Preemie | 2 | 48 | 2 |
| M.B. | 0–6 | 11 | 93 | 4 |
| M.B. | 6–12 | 5 | 43 | 3 |
| S.B. | 0–6 | 1 | 6 | 1 |
| S.B. | 0–12 | 1 | 14 | 1 |
| S.B. | 6–12 | 1 | 5 | 1 |
| A.R. | 0–6 | 1 | 6 | 1 |
| A.R. | 0–12 | 2 | 16 | 2 |
| Lac. | 0–12 | 2 | 14 | 2 |
| Hy. | 0–6 | 1 | 11 | 1 |
| Hy. | 0–12 | 2 | 11 | 2 |
| Hy. | 6–12 | 1 | 5 | 1 |
| P. Hy. | 0–6 | 1 | 12 | 1 |
| P. Hy. | 0–12 | 1 | 9 | 1 |
| P. Hy. | 6–12 | 1 | 7 | 1 |

**Supplementary Table 1:** R.T.F. = Ready to Feed; M.B. = Milk-Based; S.B. = Soy-Based; A.R. = Anti-Reflux ; Lac. = Lactose Reduced / Free; Hy. = Hydrolyzed; P.Hy. = Partially Hydrolyzed. Age in given months.

|  | **Preterm** | | **zero-six** | | **six-twelve** | |
| --- | --- | --- | --- | --- | --- | --- |
| **Types** | **Min** | **Max** | **Min** | **Max** | **Min** | **Max** |
| **R.T.F.** | 6.08 | 11.07 | - | - | - | - |
| **M.B.** | 9.65 | 12.60 | 9.44 | 26.18 | 11.03 | 18.25 |
| **A.R.** | - | - | 10.90 | 73.35 | 10.90 | 58.60 |
| **S.B.** | - | - | 46.53 | 55.64 | 46.53 | 67.98 |
| **Hy.** | - | - | 14.44 | 47.29 | 12.00 | 47.29 |
| **Lac.** | - | - | 14.61 | 53.00 | 14.61 | 53.00 |
| **P.Hy.** | - | - | 11.66 | 25.49 | 11.66 | 60.03 |

**Supplementary Table 2: Range of Mn concentration per formula category**

**Supplementary Table 2:** R.T.F. = Ready to Feed; M.B. = Milk-Based; A.R. = Anti-Reflux; S.B. = Soy-Based; Hy. = Hydrolyzed; Lac. = Lactose Reduced / Free; P.Hy. = Partially Hydrolyzed. Values given in µg/100kCAl.

**Supplementary table 3:** **Complementary feeding based on data from the Mabat Infant National Health and Nutrition Survey of 2019–2020**

| Food Item | Mean Mn  (Mg/100g) | Mean Kcal/100g | Mean Daily Grams Per Capita |
| --- | --- | --- | --- |
| Infant Formula* | - | 74 | 375 |
| Cooked Cereal | 0.17 | 98 | 123 |
| Cooked Veg | 0.31 | 81 | 114 |
| Fruits | 0.22 | 70 | 96 |
| Rice / Pasta | 0.98 | 127 | 50 |
| Fresh Vegetables | 0.12 | 81 | 37 |
| Cottage Cheese | 0.02 | 98 | 32 |
| Dairy Deserts | 0.03 | 102 | 28 |
| Potato / Sweet Potato | 0.16 | 75 | 24 |
| Bread | 0.63 | 266 | 17 |
| Yogurt | 0.00^ǂ^ | 61 | 17 |
| Poultry | 0.01 | 158 | 16 |
| Hummus | 1.06 | 243 | 14 |
| Eggs | 0.05 | 143 | 14 |
| Cooked Legumes | 1.57 | 116 | 9 |
| Beef | 0.01 | 250 | 6 |
| Dairy Drinks | 0.00^ǂ^ | 60 | 3 |
| Fish | 0.01 | 104 | 3 |
| Cookies | 0.00^ǂ^ | 500 | 2 |
| Salty Snacks | 0.00^ǂ^ | 534 | 2 |
| Chocolate Spread | 0.00^ǂ^ | 541 | 1 |
| Breakfast Cereal | 0.00^ǂ^ | 379 | 1 |
| Firm Cheeses | 0.03 | 360 | 1 |
| Butter | 0.00^ǂ^ | 717 | 0.1 |

**Supplementary Table 3:** Complementary feeding patterns were surveyed for infants aged nine-twelve months old. Data adapted from the survey. Mean Mn content was taken from the United States Department of Agriculture FoodData Central. *Data from this study. ǂ = Less than 0.01 mg/100g.
