# Supplementary material for: Manganese exposure assessment in formula-fed infants in Israel
Source: Isr J Health Policy Res. 2025 Apr 15;14:24. doi: 10.1186/s13584-025-00688-2 (PMC12001741; doi:10.1186/s13584-025-00688-2)
Supplement: Supplementary file 1 — Supplementary Material 1 [file 13584_2025_688_MOESM1_ESM.pdf]

Reviewer reports:

Overall, I appreciate the authors' attempt to address my comments. This is not an easy paper to read, and one has to wade through a lot of material before getting to the bottom-line recommendations. Nevertheless, those recommendations are now stated clearly, and I hope will be helpful to policymakers.
If the authors can make the paper more friendly for readers it would be great.

We appreciate the time and effort put into the review of the paper. However, this comment is along the line of previous comments raised by the reviewer for which we have already made extensive changes in the outline of the paper, the figures and the text to simplify our findings and make the paper more readable for audiences that are not experts in the field. Without any concreate comments we fail to see how we can address this issue any further.

I have one concern about the content: On p.6 of the manuscript the authors discuss effects of chronic overexposure of children to high levels of manganese. Although there was no attempt in this study to determine if any of the symptoms of overexposure occur in Israeli children, I sense that the authors are not concerned that significant chronic overexposure is occurring in Israeli children. If I am correct in that assumption, then it would be good for the authors to state it clearly.

We would like to point out that this comment was not previously raised. Moreover, we strongly feel there is no place to state this in the article for several reasons. First, we make no claim to quantify the total exposure to Mn, as exposure is not limited to

ingestion through the oral route. As mentioned in the background, one can be exposed to Mn through dermal absorption and through inhalation as well. Our study specifically quantifies infant exposure to Mn through ingestion of ready-to-feed formula and complementary foods only.

Consumer goods and air pollution can add to overall exposure, but these exposure routes are outside the scope of the article. Since we make no attempt to quantify total exposure, and epidemiological data are out of scope of the article, there are no scientific grounds to support the conclusion suggested by the reviewer. Therefore, stating this within the presented paper has no merit.

We have however made a short statement in line 421-425 so as to clarify that total exposure may be higher than presented within our study.

I also have an editorial concern with multiple parts:

•There are a number of minor errors in the English. Most do not alter the meaning. An example of that occurs on p.7 where there is a sentence that begins, "To evaluated Infant growth in Israel…" In proper English, it would begin, "To evaluate infant growth in Israel"….

•There is at least one example of a missing word. On p.22, the authors refer to the Codex Committee on Nutrition and Foods for Special Dietary. I believe "Uses" is missing after "Dietary".

•The authors seem to use P.V for "p-value." P.V is not a standard way of denoting p-value.

•My greatest concern is that there are many acronyms in the manuscript. I think it is OK to abbreviate labels in a complex figure, so long as there is a key to the abbreviations. The authors have done that nicely in Figure 1. But otherwise the

National Food Service
Ministry of Health
236 Yaffo St., Jerusalem
9438317 (Daniel building)
Call.habriut@moh.health.gov.il
Tel *5400 **Fax**: 02-5655969

קול הבריאות *5400

שרות המזון הארצי
משרד הבריאות
רח' יפו 236 ,ירושלים
9438317 (בניין דניאל)
Call.habriut@moh.health.gov.il
טל: 5400* **פקס:** 02-5655969

acronyms can easily slow down readers by being very hard to remember. For example, "AI" is defined on p.6, but not used again until p. 16; and TDI is also defined on p.6, but not used again until p. 17.
To handle these issues, the paper will require very careful reading and editing by a person who ideally is a native English speaker and writer and has extensive experience in writing of papers for scientific journals.

The corresponding author is a native English speaker, and both authors have extensive experience in scientific writing. This comment has only now been raised and is an editorial issue rather than a scientific one, which we feel is not grounds to reject the paper from publication. We have carefully gone through the article, proofread it, and corrected any typos and errors.
Regarding the acronyms, the article already contained a table of acronyms in the reviewed version, as per the editorial requirements of the journal. Despite this, and given the reviewer's comment, we simplified the reading by omitting several common acronyms used throughout the paper and replaced them with the full unabbreviated expressions for the benefit of the reader. These changes are not fully reflected in the track changes version submitted for review for simplicity.

Sincerely yours,
Dr. Jonatan Darr
